# 2011 Joplin, Missouri Tornado Experience, Mental Health Reactions, and Service Utilization: Cross-Sectional Assessments at Approximately 6 Months and 2.5 Years Post-Event

**DOI:** 10.1371/currents.dis.18ca227647291525ce3415bec1406aa5

**Published:** 2015-10-26

**Authors:** J. Brian Houston, Matthew L. Spialek, Jordan Stevens, Jennifer First, Vicky L. Mieseler, Betty Pfefferbaum

**Affiliations:** Disaster and Community Crisis Center, Department of Communication, University of Missouri, Columbia, Missouri, USA; Disaster and Community Crisis Center, Department of Communication, University of Missouri, Columbia, Missouri, USA; Department of Psychology, University of Missouri, Columbia, Missouri, USA; Disaster and Community Crisis Center, School of Social Work, University of Missouri, Columbia, Missouri, USA; Ozark Center, Freeman Health System, Joplin, Missouri, USA; Department of Psychiatry and Behavioral Health Sciences, University of Oklahoma College of Medicine, Oklahoma City, Oklahoma, USA

## Abstract

**Introduction.** On May 22, 2011 the deadliest tornado in the United States since 1947 struck Joplin, Missouri killing 161 people, injuring approximately 1,150 individuals, and causing approximately $2.8 billion in economic losses.

**Methods.** This study examined the mental health effects of this event through a random digit dialing sample (N = 380) of Joplin adults at approximately 6 months post-disaster (Survey 1) and a purposive convenience sample (N = 438) of Joplin adults at approximately 2.5 years post-disaster (Survey 2). For both surveys we assessed tornado experience, posttraumatic stress, depression, mental health service utilization, and sociodemographics. For Survey 2 we also assessed social support and parent report of child strengths and difficulties.

**Results.** Probable PTSD relevance was 12.63% at Survey 1 and 26.74% at Survey 2, while current depression prevalence was 20.82% at Survey 1 and 13.33% at Survey 2. Less education and more tornado experience was generally related to greater likelihood of experiencing probable PTSD and current depression for both surveys. Men and younger participants were more likely to report current depression at Survey 1. Low levels of social support (assessed only at Survey 2) were related to more probable PTSD and current depression. For both surveys, we observed low rates of mental health service utilization, and these rates were also low for participants reporting probable PTSD and current depression. At Survey 2 we assessed parent report of child (ages 4 to 17) strengths and difficulties and found that child difficulties were more frequent for younger children (ages 4 to 10) than older children (ages 11 to 17), and that parents reporting probable PTSD reported a greater frequency of children with borderline or abnormal difficulties.

**Discussion.** Overall our results indicate that long-term (multi-year) community disaster mental health monitoring, assessment, referral, outreach, and services are needed following a major disaster like the 2011 Joplin tornado.

## Introduction

On May 22, 2011, “one of the most devastating tornadoes” in U.S. history struck Joplin, Missouri 1 killing 161 people and injuring approximately 1,150 individuals 2. The tornado was rated an EF-5 on the Enhanced-Fujita Scale (the maximum rating) with wind speeds over 220 miles per hour 1, and resulted in a damage path that was 22.1 miles long and up to 1 mile wide 1. The tornado struck the core of Joplin and destroyed approximately one-third of all houses as well as many businesses and schools 3 ^,^ 4 , resulting in $2.8 billion of economic losses 5. Overall, in just one afternoon, the 2011 Joplin tornado resulted in almost three times the average annual number of tornado fatalities for the entire U.S. 2.

Beyond the physical, environmental, and economic toll of the Joplin tornado, catastrophic disasters such as this event cause mental health problems for individuals who experience the disaster directly or live in the affected community. Research on disaster mental health has most frequently examined the association between disaster experience and posttraumatic stress disorder (PTSD) or PTSD symptoms 6. In a systematic review of natural disaster studies, Neria and colleagues 7 found that the prevalence of PTSD among individuals directly experiencing a disaster ranged from approximately 30% to 40%, while prevalence of PTSD in the general population where a disaster occurred ranged from approximately 5% to 10%. Across studies, PTSD has been found to be greater for individuals with more severe exposure to a disaster 7. Major depression also may occur following a disaster affecting up to approximately 25% of directly exposed disaster survivors 8. In addition to adults, children and youth are also at risk for PTSD or depression following a disaster 6 ^,^ 9. In a review of 160 disaster studies, Norris and colleagues 6 concluded that youth were at greater risk than adults for mental health problems following an event. Overall, young people’s reactions to a disaster “generally parallel those of their parents in degree” 10.

While tornadoes occur frequently in North America with over 1,000 on average per year in the United States 11 , research on the mental health effects of tornadoes is less extensive than research on some other types of disasters (e.g., hurricanes). Two early studies of tornado mental health identified a large range of PTSD prevalence post event. On the low end, in a study of 42 adult survivors of a 1988 tornado in northern Florida, conducted within 1 month after the event, North and colleagues 12 identified only 1 adult (2.4%) with PTSD related to the tornado and only 3 adults (7.1%) with post-event onset of depression. Conversely, in a study of 116 adult survivors 5 months after a 1984 severe tornado in a rural part of eastern North Carolina, Madakasira and O’Brien 13 found that 59% of survivors met criteria for PTSD. Both of these studies assessed DSM-III PTSD using primarily in-person interviews with adults affected by the tornado who were recruited using victim lists and door-to-door visits. Because of similarities in research approaches, differences in the severity of these two tornadoes may explain the range of post-event PTSD prevalence identified in these two studies. For example, the Florida tornado studied by North and colleagues 12 destroyed or severely damaged approximately 100 "structures" (including nonresidential buildings), killed 4 people, and injured 17. Conversely, the North Carolina tornado examined by Madakasira and O'Brien 13, damaged approximately 450 "dwelling units," killed 9 people, and injured over 150. Thus because the tornado in North Carolina was apparently more severe (in terms of damage, death, and injury) it may have resulted in greater PTSD prevalence among residents. Additionally, North and colleagues 12 conducted their assessment at approximately one month post-disaster while Madakasira and O'Brien 13 assessed later (at approximately 5 months post-disaster), and the later time frame may have captured a greater PTSD prevalence that developed over time.

More recent studies, though still few in number, have identified a smaller range of post-tornado PTSD prevalence. In a study of 105 adults seeking healthcare in a rural medical clinic approximately 1 year after multiple severe tornadoes struck rural southern Minnesota in 1998, Polusny and colleagues 14 found that approximately 14.6% of adult participants met diagnostic criteria for PTSD. Polusny and colleagues 15 also assessed 288 adolescents and their parents who were exposed to severe tornadoes in rural southern Minnesota in 1998 and found that approximately 18% of parents evidenced probable PTSD. In a chart abstraction of 1,398 hospital patients and a case-control telephone survey of 298 injured and uninjured control participants following the April 2011 tornadoes in Alabama, Niederkrotenthaler and colleagues 16 found that 22.1% of participants screened positive for PTSD symptoms.

A few research studies have also examined the effect of tornado experience on child and adolescent mental health. In a study of 152 children (age 6 to 12 years) living in rural Oklahoma who experienced a severe tornado in 1999, Evans and Oehler-Stinnett 17 found that 25% of children met stringent criteria for a PTSD diagnosis. In the study by Polusny and colleagues 15 of 288 adolescents and their parents who were exposed to severe tornadoes in rural southern Minnesota in 1998, the researchers found that approximately 13% of adolescents (age to 12 to 19 years) evidenced probable PTSD. Lack and Sullivan 18 assessed 102 children (age 8 to 12 years) in rural Oklahoma 13 months after experiencing strong tornadoes and found that 20.5% of the children exhibited severe or very severe posttraumatic stress symptoms (PTSS). Finally, in a study of 2,000 Alabama and Joplin, Missouri adolescents (age 12 to 17 years) and their caregivers who experienced the 2011 tornadoes, Adams and colleagues 19 found that at between 4 to 13.5 months post-disaster, 6.7% of adolescents (age 12 to 17 years) met criteria for PTSD and an estimated 7.5% met criteria for a major depressive episode (MDE). Within these child and adolescent tornado studies, female participants have been found to exhibit more PTSD and depression than males 15 ^,^ 19;more disaster exposure (in terms of perceived life threat or of experiencing an injury to a caretaker) was associated with more PTSD 15 ^,^ 19; and younger adolescents have been found to have more parent-reported PTSD compared to older adolescents 15.

The current study seeks to add to the knowledge base addressing the mental health impact of tornadoes by assessing the effects of “the deadliest single tornado to hit the United States since modern recordkeeping began in 1950” 2. We assess Joplin adults at two times (approximately 6 months and 2.5 years post-event) with two separate cross-sectional surveys. For both surveys we assess tornado experience, posttraumatic stress, depression, mental health service utilization, and sociodemographics. For the second survey we also assess social support and parent report of child strengths and difficulties.

## Materials and Methods

We conducted two surveys of Joplin, Missouri residents to examine their 2011 tornado experience, mental health reactions, and service utilization following the event. Survey 1 was conducted approximately 6 months after the tornado and Survey 2 was conducted approximately 2.5 years after the event. Both surveys are described below.

**Survey 1**

Survey 1 involved a random digit dialing (RDD) sample (N = 380) of residents in the Joplin, Missouri city limits. This survey was conducted approximately 6 to 7 months after the May 22, 2011 tornado (the survey was fielded from January 4 to 22, 2012) Participants were eligible for the study if they were at least 18-years-old and were residents of Joplin, Missouri at the time of the 2011 tornado. The survey was conducted by trained interviewers from the University of Missouri’s Health & Behavioral Risk Research Center using a Computer Assisted Telephone Interviewing (CATI) system. Interviewers used the RDD sample to contact households, determine eligibility, and describe the study. Qualifying participants were required to provide verbal consent before participating in the survey. The verbal consent process and all study procedures were reviewed and approved by the University of Missouri Institutional Review Board (IRB). The response rate (RR1; the number of completed interviews divided by the number of contacts made or attempted and not determined to be ineligible) for the survey was 33% and the cooperation rate (COOP1; the number of completed interviews divided by the number of eligible respondents contacted) was 81% 20.

Measures

*Tornado Experience*

Tornado experience was assessed using items from other disaster studies that assessed the extent of disaster experience and impact 21 ^,^ 22, and were adapted for the 2011 Joplin tornado. Participants were asked whether they took shelter during the tornado, whether they saw the tornado, whether they heard the tornado, and whether they were injured during the tornado, with possible responses of yes or no for each item. Participants were also asked what impact the tornado had on their home, with possible responses including none, mild damage, moderate damage, severe damage, and totally destroyed. For analysis, the home damage responses were recoded into no damage and damage. Participants were also asked if any of their friends, colleagues, or neighbors died in the tornado and if any of their family members or relatives died in the tornado, with possible responses of yes or no.

Responses to the 7 tornado experience questions were also summed resulting in a total score that ranged from 0 to 7, and the total scores were then recoded so that total scores of 0 to 1 indicated low impact, total scores of 2 or 3 indicated moderate impact, and scores of 4 to 7 indicated high impact.

*Posttraumatic Stress*

Posttraumatic stress was assessed using the Trauma Screening Questionnaire (TSQ) 23, a brief 10-item self-report measure created to screen for posttraumatic stress disorder (PTSD). The TSQ has been validated on independent samples within one year of a traumatic event 24. In a study with train crash survivors, the instrument was found to have sensitivity of .86, specificity of .93, and overall efficiency of .90 24. Versions of the TSQ have been used in studies of Hurricane Katrina survivors and Virginia Tech students following the mass campus shooting in 2007 25 ^,^ 26. The TSQ’s 10-items include PTSD reactions such as “feeling upset by reminders of the event” and “being jumpy or being startled at something unexpected.” Participants were instructed to consider their personal reactions to the May 22, 2011 Joplin tornado and to indicate whether or not they had experienced each item at least twice in the past week. If participants indicated they had experienced an item at least twice in the past week the response was coded as 1; if not the response was coded as 0. For analysis, responses were summed and participants who had experienced six or more of the items were coded as experiencing probable PTSD related to the tornado while participants experiencing fewer than six items were coded as not experiencing probable PTSD related to the tornado 23.

*Depression*

Depression was assessed using the two items from the Patient Health Questionnnaire-2 depression subscale (PHQ-2) 27. These questions asked “Over the last 2 weeks, how often have you found that you had little interest or pleasure in doing things?” and “Over the last 2 weeks, how often have you felt down, depressed, or hopeless” with possible response options of 0 (not at all), 1 (several days), 2 (more than half the days), and 3 (nearly every day). Responses to both questions were summed and total scores of 3 or more were coded as representing current depression 27.

*Mental Health Service Utilization*

Mental health service utilization was assessed by asking participants how often they had engaged in the following activities: talked to a counselor or mental health professional about the tornado and talked to a minister, priest, rabbi, or other religious professional about the tornado with possible response options ranging from 1 (not engaged in that activity) to 7 (engaged in that activity frequently). For analysis, responses of 1 or 2 were recoded to represent no or little mental health service utilization; responses of 3, 4, or 5 were recoded to represent moderate mental health service utilization; and responses of 6 or 7 were recoded to represent frequent mental health service utilization.

*Sociodemographics*

Participants also reported their gender, age, and education level.

**Survey 2**

Survey 2 involved an online survey of Joplin adults (N = 438). This survey was conducted approximately 2.5 years after the May 22, 2011 tornado (the survey was fielded from September 30 to December 13, 2013). Participants were eligible for the study if they were at least 18-years-old and were residents of Joplin, Missouri at the time of the 2011 tornado. Participants were recruited with flyers posted and distributed in locations in Joplin, Missouri such as medical facilities and the public library. Recruitment advertisements for the study were posted on Facebook pages related to the Joplin tornado and Facebook advertisements were also purchased so that the advertisements appeared on the feed of some Facebook users in the Joplin, Missouri area. All study recruitment materials included the Internet address that participants could use to access and complete the survey, which was designed in and hosted by Qualtrics online survey software. When participants visited the online survey they first read a consent form and were required to click a button to indicate their consent to participate in the study. The opportunity to participate in an iPad drawing was described in survey recruitment materials, and one iPad was given to a randomly selected survey participant following the drawing. Participants choosing to enter the iPad drawing provided their name, email address, and phone number following the survey and this information was stored in a separate database from the main study. The online consent process, iPad giveaway, and all study procedures were reviewed and approved by the University of Missouri Institutional Review Board (IRB). Overall, 565 participants provided consent and began the study and 127 participants exited the survey before completing at least half of the survey questions, resulting in a breakoff rate of 22.48%. Participants who broke off from the survey were not included in the final analysis, resulting in a final sample of N = 438.

Measures

For Survey 2, we assessed tornado experience, posttraumatic stress, depression, social support, mental health service utilization, parent report of child strengths and difficulties, and sociodemographics.

*Tornado Experience*

Tornado experience was assessed using the same seven questions used in the first survey. See Survey 1 for a description of these items.

*Posttraumatic Stress*

Posttraumatic stress was measured using 22 items from the Impact of Events Scale—Revised (IES-R) 28. The IES-R lists a series of difficulties, and participants were instructed to report how distressing or bothersome each difficulty had been within the past week with respect to the May 22, 2011 tornado. Responses ranged from 0 (not at all) to 4 (extremely). Sample items include: “Any reminder brought back feelings about it,” “I felt irritable and angry,” and “My feelings about it were kind of numb.” Responses to all questions were summed and a cumulative score of 33 or above was coded as probable PTSD and a cumulative score of less than 33 was coded as no PTSD 29.

*Depression*

Depression was assessed using the two depression items from the Patient Health Questionnnaire-2 subscale (PHQ-2) 27. See Survey 1 for a description of this measure and scoring process.

*Social Support*

Social support was assessed using the 12-item Multidimensional Scale of Perceived Social Support 30. This scale includes items such as “There is a special person who is around when I am in need,” “My family really tries to help me,” “I have friends with whom I can share my joys and sorrows,” "There is a special person in my life who cares about my feelings," "I get the emotional help and support I need from my family," and "I can talk about my problems with my friends." Response options range from 1 (very strongly disagree) to 7 (very strongly agree). Responses to all questions were summed and total scores of 12-48 were coded as low social support, total scores of 49-68 were coded as moderate social support, and total scores of 69-84 were coded as high social support 30.

*Mental Health Service Utilization*

Mental health service utilization was assessed by asking participants how often they had talked to a counselor or mental health professional about the tornado and talked to a minister, priest, rabbi, or other religious professional about the tornado in the last few months with possible response options ranging from 1 (never) to 5 (a great deal). For analysis, responses of 1 or 2 were recoded to no or little mental health service utilization, responses of 3 were recoded to moderate mental health service utilization, and responses of 4 or 5 were recoded frequent mental health service utilization. These questions were the same as used in Survey 1, though for Survey 1 the response options were based on a 7-point scale and for Survey 2 the response options were based on a 5-point scale.

*Child Strengths and Difficulties*

Participants were asked if they had a child between the ages of 4 and 17-years-old. Participants indicating they had a child in this age range (n = 114) were asked to report the age of their child and if participants had more than one child they were instructed 31 to pick just one of their children. Participants reported their child’s age and were then shown questions from the Strengths and Difficulties Questionnaire (SDQ) . The SDQ includes 25 questions that assess parent report of child emotional symptoms, conduct problems, hyperactivity/inattention, peer relationship problems, and prosocial behaviors 31. Participants were instructed to consider their child’s behavior over the last six months and indicate whether each item on the SDQ was 0 (not true), 1 (somewhat true), or 2 (certainly true) of their child. Example items include “considerate of other people’s feelings;” “many fears, easily scared,” and “often complains of headaches, stomach-aches or sickness.” The SDQ is available in versions for children age 4 to 10 and age 11 to 17, and respondents were shown the appropriate version of the SDQ based on the reported age of their child. The SDQ measure was created by summing all items and total scores of 0-13 were coded as normal, total scores of 14-16 were coded as borderline, and total scores of 17-40 were coded as abnormal 31.

*Sociodemographics*

Participants also reported their gender, age, and education level.

**Analysis**

Data from both surveys were weighted to match age and gender distributions from the 2010 census data for Joplin, Missouri. Missing data were calculated using multiple imputation in SPSS (Version 22.0), a procedure that uses chained equations imputation to create a specified number of complete data sets that can then be pooled and used in analyses 32. In this case, five complete datasets were created via multiple imputation and pooled for all analyses. All data analysis was conducted using SPSS (Version 22.0). Frequency distributions were calculated for most variables. Frequency distributions for tornado experience, probable PTSD, and current depression from Survey 1 and Survey 2 were compared using chi-square tests. Cross tabulations were calculated between probable PTSD, current depression, and mental health service utilization; and probable PTSD, current depression, and child SDQ. Cross tabulations were tested using chi-square tests.

Logistic regression models were calculated to predict probable PTSD and current depression for both surveys. These logistic regression models included gender, age, education, and tornado impact as predictors. The models for Survey 2 also included social support. For regression analysis, age was recoded into a categorical variable in which participants age 18 to 45 were coded as younger and participants older than 45 were coded as older. Education was also recoded, so that “grade school or less (0-8 grade)” and “some high school” were coded as low education; “high school graduate” and “some college” were coded as medium education; and “college graduate” or “graduate degree” were coded as high education. Responses to the 7 tornado experience questions were summed resulting in a total score that ranged from 0 to 7, and the total scores were then recoded so that total scores of 0 to 1 indicated low impact, total scores of 2 or 3 indicated moderate impact, and scores of 4 to 7 indicated high impact. For Survey 2, the three levels of social support (low, medium, high), as described previously, were also included in the model.

## Results

Results for both surveys are presented together. See Table 1 for demographic characteristics of participants from both surveys.

**Figure:**
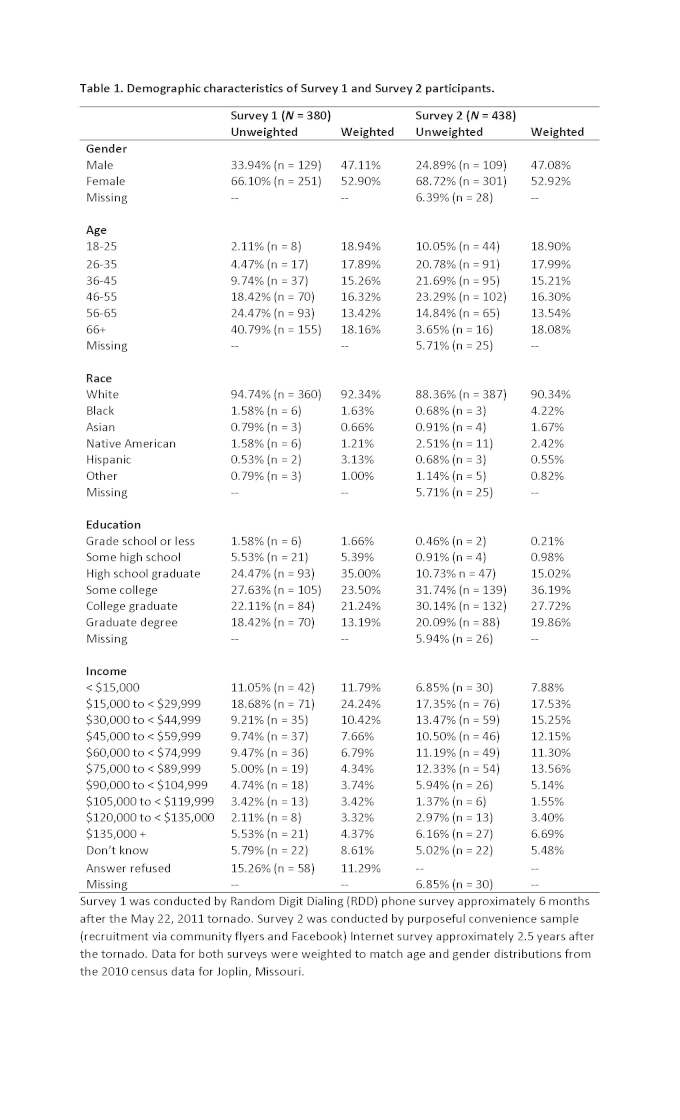
**Table 1. Demographic characteristics of Survey 1 and Survey 2 participants.**

*Tornado Experience*

See Table 2 for results related to the seven questions on tornado experience. For both surveys, over half of respondents took shelter from the tornado and heard the tornado. Many participants in both surveys (Survey 1: 42.37%; Survey 2: 56.62%) knew a friend or neighbor who was killed in the tornado. A greater proportion of participants in Survey 2 saw the tornado, experienced damage to their home, and were injured in the tornado.

**Figure:**
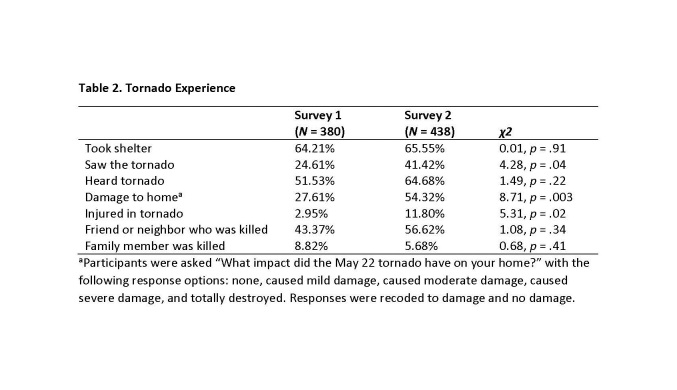
**Table 2. Tornado Experience**

Responses to the seven tornado items were also summed resulting in a total tornado impact score that ranged from 0 to 7. These scores were then recoded so that scores of 0 or 1 were considered low tornado impact, scores of 2 or 3 were considered moderate impact, and scores of 4 through 7 were considered high impact. For Survey 1, 37.26% of participants experienced low tornado impact, 42.39% experienced moderate impact, and 20.32% experienced high impact. For Survey 2, 18.93% of participants experienced low impact, 38.72% experienced moderate impact, and 42.35% experienced high impact. A chi-square test of the tornado impact frequency distributions was statistically significant (χ2 = 13.89, p < .001), indicating that participants in Survey 2 experienced greater tornado impact than did participants in Survey 1.

*Posttraumatic Stress and Depression*

For Survey 1, 12.63% of participants evidenced probable PTSD (assessed using the TSQ), while for Survey 2, 26.74% of participants evidenced probable PTSD (assessed using the IES-R). The chi-square test examining these distributions was significant (χ2 = 5.06, p = .02), indicating a greater proportion of Survey 2 participants reported probable PTSD. Depression was measured using the PHQ-2 for both surveys. For Survey 1, 20.82% of participants reported current depression, while for Survey 2, 13.33% of participants reported current depression. The chi-square test examining these distributions was not statistically significant (χ2 = 1.98, p = .16).

Logistic regression models were calculated with probable PTSD and current depression as the dependent variables. Predictor variables included gender, age, education, and tornado impact for both surveys, and also included social support for Survey 2. See Table 3 for results. All interpretations of predictors are made with all other predictors held constant. For both surveys, females were more likely than males to report probable tornado PTSD (Survey 1, odds ratio [OR], 2.26; 95% confidence interval [CI]: 1.03-4.96; Survey 2, OR, 2.64; 95% CI, 1.49-4.68). For Survey 1, females were less likely than males to report current depression (OR, 0.48; 95% CI, 0.26-0.87). For both surveys, older participants were less likely than younger participants to report current depression (Survey 1, OR, 0.54; 95% OR, 0.29-0.99; Study 2, OR, 0.47; 95% CI, 0.26-0.88). For Survey 1, participants with medium or high levels of education reported less probable tornado PTSD and current depression than did participants with low levels of education (See Table 3). There was no difference between level of education and probable tornado PTSD or current depression for Survey 2.

Participants experiencing high tornado impact were more likely than participants experiencing low tornado impact to report probable PTSD for both surveys and more likely to report current depression for Survey 1 (See Table 3). Social support was only assessed in Survey 2, and the logistic regression model indicated that participants with high levels of social support were less likely than participants with low levels of social support to report probable PTSD (OR, 0.36; 95% CI, 0.18-0.73) and current depression (OR, 0.35; 95% CI, 0.17-0.75).

**Figure:**
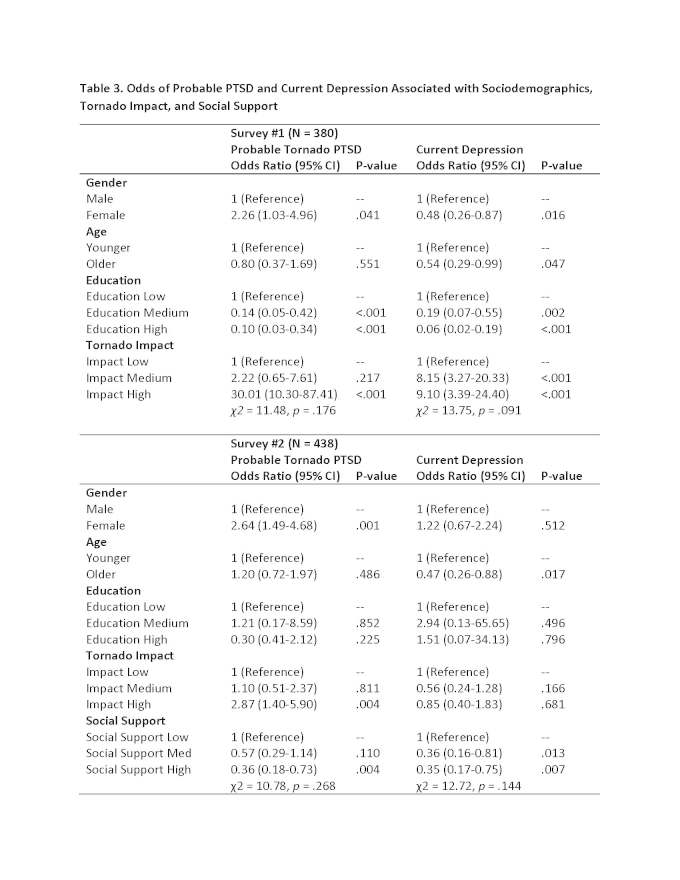
**Table 3. Odds of Probable PTSD and Current Depression Associated with Sociodemographics, Tornado Impact, and Social Support**

*Mental Health Service Utilization*

See Table 4 for results related to mental health service utilization. For both surveys, most respondents overall (84.66% to 92.6%) reported that they had talked to mental health or religious professionals about the tornado not at all or very little. For Survey 1, participants with probable PTSD were more likely than respondents without probable PTSD to often talk with a mental health or religious professional. However, the majority of participants with probable PTSD at Survey 1 reported no or very little communication with mental health (66.67%) or religious (70.21%) professionals. These patterns were also largely evident for current depression during Survey 1. While approximately one-third (34.01%) of participants at Survey 1 with current depression reported often speaking with a counselor or mental health professional, the majority (64.74%) of participants with current depression at Survey 1 had spoken with a counselor or mental health professional not at all or very little.

Interaction with mental health and religious professionals was less frequent at Survey 2. The vast majority of individuals with probable PTSD reported speaking with a counselor or mental health professional (83.42%) and a religious professional (85.91%) not at all or very little. These rates were similar for participants with current depression at Survey 2 (see Table 4).

**Figure:**
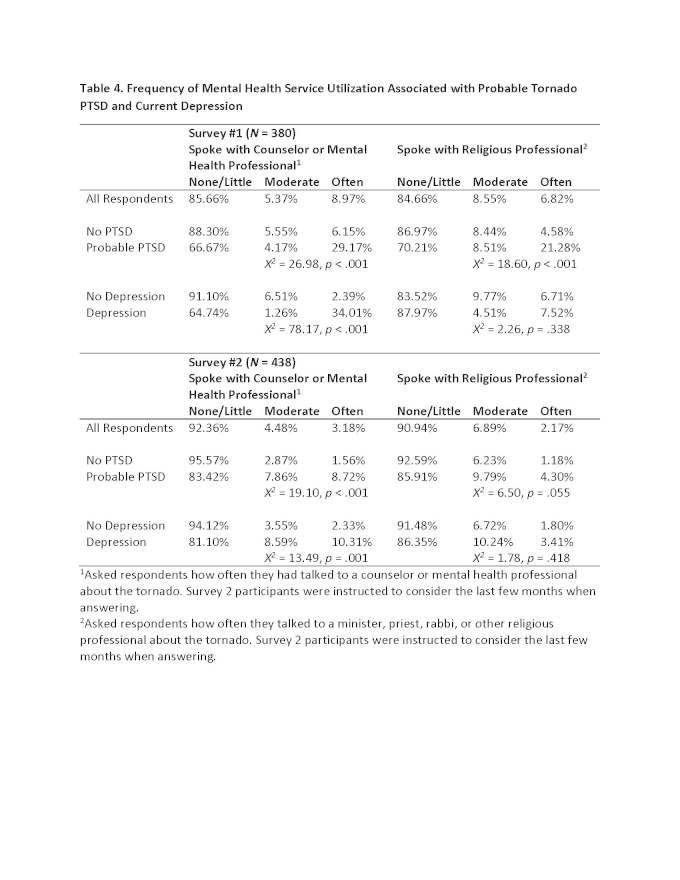
**Table 4. Frequency of Mental Health Service Utilization Associated with Probable Tornado PTSD and Current Depression**

*Parent Report of Child Strengths and Difficulties*

For Survey 2, 114 participants indicated that they had a child between the ages of 4 and 17. See Table 5 for overall SDQ results for younger children (age 4-10), older children (age 11-17), and all children. For younger children, over half (57.62%, n = 34) indicated borderline or abnormal SDQ scores based on parent report, with over one-quarter indicating abnormal behavior (28.81%). The rates were lower for older children, as 30.91% of youth age 11-17 were determined to have borderline or abnormal behavior based on parent report.

**Figure:**
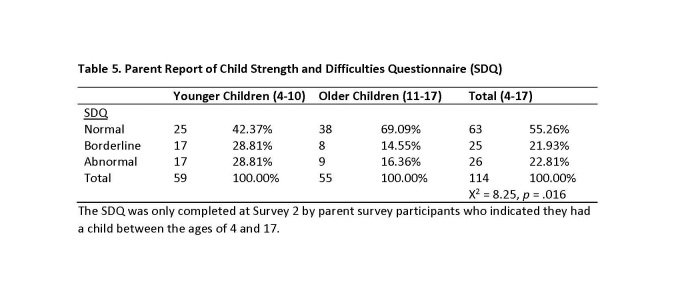
**Table 5. Parent Report of Child Strength and Difficulties Questionnaire (SDQ)**

Table 6 presents results of parent report of child SDQ and participants’ report of probable tornado PTSD and current depression. Participants who reported probable tornado PTSD reported higher rates of borderline (27.03%) and abnormal (37.84%) child behavior than did participants who did not report probable tornado PTSD (borderline, 19.23%; abnormal, 16.67%). Participants reporting current depression also reported higher rates of borderline (33.33%) and abnormal (33.33%) child behavior than did participants not reporting current depression (borderline, 20.79%; abnormal, 20.79%).

**Figure:**
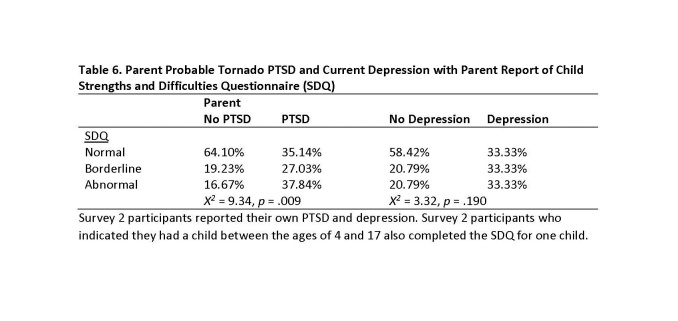
**Table 6. Parent Probable Tornado PTSD and Current Depression with Parent Report of Child Strengths and Difficulties Questionnaire (SDQ)**

## Discussion

We surveyed Joplin adults at approximately 6 months and 2.5 years after the deadliest tornado in the U.S. since modern recordkeeping began 2. Survey 1 utilized a RDD telephone sample and Survey 2 utilized a purposeful convenience sample and was conducted online.

*Tornado Experience*

Participants from both of our samples reported significant 2011 tornado experience. For both surveys a majority of participants reported taking shelter from the tornado and hearing the tornado. Many participants from both surveys knew a friend or neighbor who was killed in the tornado (Survey 1, 43.37%; Survey 2, 56.62%). Overall, more Survey 2 participants reported seeing the tornado (Survey 1, 24.61%; Survey 2, 41.42%), experiencing damage to their home (Survey 1, 27.61%; Survey 2, 54.32%), and being injured in the tornado (Survey 1, 2.95%; Survey 2, 11.80%). Additionally, when we created an overall index of tornado experience, more Survey 2 participants (42.35%) reported high overall tornado impact than did Survey 1 participants (20.32%). In all, participants in both studies reported tornado experience that largely matched a convenience sample survey of 141 Joplin adults conducted 1 to 9 months post-event that found that 49% of participants reported damage to their home or possessions, 18% reported injuries from the tornado, and 69% reported taking shelter 2.

The somewhat greater tornado experience for our Survey 2 participants may be related to our data collection approaches. Our Survey 1 sample, while a probability sample, only included landline phone numbers and did not include cell phones. Relying on landlines only may not be optimal in normal conditions (due to an increasing population of cell phone only households) 33 , and in post-disaster contexts this may be additionally problematic. This is because at approximately six months post-tornado (when our Survey 1 was fielded), many individuals who experienced the most home damage (and thus were likely to have considerable tornado experience) might have still been living in temporary housing (e.g., rented apartments or houses) and thus may not have established landline services in that temporary housing. Thus it is possible our Survey 1 results somewhat underestimate the population tornado impact. At the same time, because Survey 2 used a convenience sample, individuals who responded to the survey recruitment flyers and advertisements may have been more likely to have had significant tornado experience or to still be coping with the tornado 2.5 years later (when Survey 2 was conducted). Therefore our Survey 2 may have overestimated population impact. Our tornado experience results are in line with expectations based on the nature of the event and other research on the Joplin tornado 2, but overall could be strengthened with better sampling. We did weight our analysis for both surveys to match Joplin 2010 census distributions of age and gender, giving us additional confidence in the results.

*Posttraumatic Stress and Depression*

For Survey 1, 12.6% of participants indicated probable PTSD and for Survey 2 the rate increased to 26.74%. The difference in these rates may be attributable in part to the data collection approaches previously described or to differences in instruments used to assess probable PTSD (the TSQ in Survey 1 and the IES-R in Survey 2). Both of these instruments are brief screening measures that include cut-off scores to determine no PTSD or probable PTSD for each individual case. While such approaches are often used in disaster mental health epidemiological research 7 ^,^ 25 ^,^ 26 ^,^ 34 and have proven reliable 24 ^,^ 28 ^,^ 29 ^,^ 36, some researchers have argued that face-to-face clinical assessment is necessary for reliable measurement of PTSD prevalence (see Neria and colleagues 7 for discussion).

Another possible explanation for these differing rates is that although PTSD rates typically decline in a population following a natural disaster 7, the historic amount of death, injury, and destruction resulting from the Joplin tornado coupled with challenges in rebuilding individual homes and businesses as well as a large portion of the entire city of Joplin may have resulted in a non-typical situation wherein population rates of PTSD increased from 6 months to 2.5 years post-event. Ideally, a state and federal disaster mental health response following a tornado (coupled with individual, family, and community resilience capacities) would result in declining rates of PTSD in the months and years after the event, but the effectiveness of these processes in meeting community need cannot be assumed.

Additionally, even if our Survey 2 rate of probable PTSD (26.74%) is slightly overestimated as a result of convenience sampling, the rate for this sample alone translates into a significant number (n = 117) of self-selected individuals potentially living with PTSD several years after the event. Thus this result indicates a significant need for a clinical assessment and possible treatment for PTSD and provides support for the use of periodic PTSD screening over time post event. In contrast to PTSD prevalence, current depression rates were not statistically different for Survey 1 (20.82%) and Survey 2 (13.33%). Persistent depression is not surprising given the magnitude of destruction caused by this tornado. In addition to the possibility that for some respondents depression represented recurrence of pre-event psychopathology, post-event depression may occur secondary to unresolved trauma or PTSD and/or to enduring adversity that takes a toll over time.

In our regression models, for both samples women were more likely (Survey 1, OR, 2.26, 95% CI, 1.03-4.96; Survey 2, OR, 2.64, 95% CI, 1.49-4.68) than men to report probable PTSD. Women reporting higher rates of PTSD post-disaster is a common finding in the disaster mental health literature 6 ^,^ 34 . Conversely, for Survey 1 men were more likely to report current depression than women (OR, 0.48, 95% CI, 0.26-0.87), which is an unusual result. The Joplin tornado damaged and destroyed numerous businesses and thus affected the employment of many individuals living in Joplin 4 ^,^ 5. This employment and economic impact coupled with clean-up and rebuilding challenges may have contributed to a higher rate of depression among Joplin men at 6 months, though this explanation is speculative. For both studies older participants were less likely (Survey 1, OR, 0.54, 95% CI, 0.29-0.99; Survey 2, OR, 0.47, 95% CI, 0.26-0.88) than younger participants to report current depression. Thus in terms of depression, older adults illustrated resilience at 6 months and 2.5 years post-disaster compared to younger adults 37. Participants with moderate and high levels of education were less likely than participants with low levels of education to indicate probable PTSD and current depression at Survey 1 (Moderate, OR, 0.14, 95% CI, 0.05-0.42; High, OR, 0.10, 95% CI, 0.03-0.34), but not at Survey 2. Participants reporting high tornado impact reported more probable PTSD than those reporting low tornado impact for both surveys (Survey 1, OR, 30.01, 95% CI, 10.30-87.41; Survey 2, OR, 2.87, 95% CI, 1.40-5.90), and reported more current depression at Survey 1 (OR, 9.10, 95% CI, 3.39-24.40). Finally, participants with high levels of social support (assessed only at Survey 2) reported less probable PTSD (OR, 0.36, 95% CI, 0.18-.073) and current depression (OR, 0.35, 95% CI, 0.17-0.75) than did participants with low levels of social support. Overall then, we found that gender, disaster exposure, education, and social support all help explain trends in mental health following the Joplin tornado, and these results largely fit with the existing disaster literature 6 ^,^ 7 ^,^ 34 ^,^ 38.

*Mental Health Service Utilization*

We assessed mental health service utilization at Surveys 1 and 2 with two questions that asked participants how often they spoke with mental health or religious professionals about the tornado. Survey 2 participants were instructed to consider the last few months. For all participants at both surveys, most respondents (84.66% to 92.36%) reported speaking to mental health or religious officials not at all or very little. For both surveys, participants indicating probable PTSD or current depression reported more service utilization, but the rates were still low and declined from Survey 1 to Survey 2. For example, at Survey 1, 66.67% of participants indicating probable PTSD reported speaking with a mental health professional not at all or very little, and 70.21% reported speaking with a religious professional not at all or very little. Therefore, six months after the tornado the majority of participants with probable PTSD were receiving none or very little mental health services. The rates for participants with current depression at Survey 1 were similar. For Survey 2, the proportion of participants with probable PTSD speaking with mental health professionals (83.42%) or religious professionals (85.91%) not at all or very little increased. Again, at Survey 2 the results for depression were similar.

These results illustrate an early (6 month post-event) and sustained (2.5 years post-event) need for mental health service utilization following the 2011 Joplin tornado. Thus more research is needed to understand why individuals do or do not utilize mental health services following a catastrophic disaster like the Joplin tornado. The mental health service system was directly affected by this tornado in that mental health providers living in Joplin were personally affected and Joplin health care facilities were damaged and destroyed 39. This meant that the pre-tornado mental health service system had reduced capacity immediately following the disaster. At the same, time there was influx of state and federal support for mental health programs, information, and outreach such as the U.S. Substance Abuse and Mental Health Services (SAMHSA) and Federal Emergency Management Agency (FEMA) Crisis Counseling Assistance and Training Program (CCP) [http://www.samhsa.gov/dtac/ccp] and the SAMHSA Emergency Response Grant (SERG) [http://www.samhsa.gov/disaster-preparedness/samhsas-efforts]. A Missouri Department of Mental Health 39 report indicates that between May 2011 and September 2012 over 237,000 brief crisis counseling educational or supportive contacts occurred and over 47,000 mental health service referrals were made. Additionally Ozark Center’s Access Crisis Intervention center experienced more than a 100% increase crisis call volume in the month after the tornado compared to the previous year (June 2010, 415 calls; June 2011, 1075 calls). Thus a significant amount of mental health outreach and referral was evident in the 1.5 years following the tornado.

Overall though it is simply not known if this Joplin post-disaster mental health outreach and corresponding service capacity was sufficient given the need. Moreover, if the service system capacity was sufficient to address the community mental health need, then other barriers (e.g., individual attitudes about mental health services, social stigma, financial and transportation issues, awareness of mental health programs) may have prevented individuals with mental health need from seeking, utilizing, or maintaining services post-disaster. Also, some Joplin residents may have had contact with representatives from the CCP program and not considered those interactions to involve mental health professionals when answering our survey questions. Such perceptions are largely by design, as the Joplin CCP generally employed paraprofessionals (not mental health providers) and provided a range of non-counseling services such as supportive listening, help with problem solving, psychoeducation, and public information. Thus a broader assessment of the disaster system of care is needed to fully describe the use of services following an event, and such an assessment approach applied to Joplin might indicate higher service utilization. Ultimately, much more research on post-disaster mental health service provision, seeking, utilization, and outreach is needed.

*Child Strengths and Difficulties*

At Survey 2 we asked participants with a child between the ages of 4 and 17 (n = 114) to report on their child’s strength and difficulties using the SDQ 31. Responses were scored and coded as normal, borderline, or abnormal difficulties. We found that a greater proportion of parents of younger children (ages 4-10) reported borderline or abnormal child difficulties (57.62%) than did parents of older children (ages 11-17; 30.91%). More than one-fourth (28.81%) of the younger children were reported by a parent as having abnormal difficulties at Survey 2. We also examined how parent probable PTSD status was related to parent report of child strengths and difficulties. We found that parents with probable PTSD were more likely to report their children as having borderline (no PTSD, 19.23%; probable PTSD, 27.03%) and abnormal (no PTSD, 16.67%; probable PTSD, 37.84%) difficulties. Therefore we find evidence to support previous research that parents with post-disaster difficulties often have children who are experiencing similar difficulties 10. The explanation for the similarity between parent and child disaster reactions is not clear. These similarities may be the result of comparable disaster experience among members of the same family or shared biological bases for reactions to an event 40. Distress in parents may signal danger and generate a sense of insecurity in children 10. Young children are especially likely to be influenced by their parents’ reactions and by their interactions with their parents because of their limited cognitive ability to conceptualize the event 41 and their greater dependence on their parents 42.

## Conclusions

We conducted two surveys of Joplin, Missouri residents to examine 2011 tornado experience, mental health reactions, and service utilization following the event. Survey 1 was conducted approximately 6 months after the tornado and utilized an RDD sample. Survey 2 was conducted approximately 2.5 years after the event and utilized a purposive convenience sample and was conducted online.

Probable PTSD prevalence was 12.63% at Survey 1 and 26.74% at Survey 2, while current depression prevalence was 20.82% at Survey 1 and 13.33% at Survey 2. The increase of probable PTSD from Survey 1 to Survey 2 may be related, in part, to differing survey sample strategies. However, the Survey 2 probable PTSD prevalence rate indicates an ongoing community mental health need at 2.5 years post-tornado.

Less education and more tornado experience was generally related to a greater likelihood of experiencing probable PTSD and current depression in both surveys. Women were more likely to report probable PTSD at both surveys. Men and younger participants were more likely to report current depression at Survey 1. Low levels of social support (which was assessed only at Survey 2) were found to be related to more probable PTSD and current depression 2.5 years after the tornado.

We found low rates of mental health service utilization for all participants in both surveys. Mental health service utilization rates were also low for participants reporting probable PTSD and current depression. Moreover, these utilization rates declined from Survey 1 to Survey 2. At Survey 2 parents of children age 4 to 17 also reported child strengths and difficulties. We found that child difficulties (borderline and abnormal) were more frequent for younger children (ages 4 to 10) than older children (ages 11 to 17). We also found that parents indicating probable PTSD reported a greater frequency of children with borderline or abnormal difficulties more than two years after the event.

Overall, following the catastrophic 2011 Joplin, Missouri tornado we found a significant adult mental health need at 6 months and 2.5 years post-disaster. Additionally at 2.5 years post-disaster we see that mental health difficulties experienced by parents are echoed in parent assessment of their children. Our results indicate that long-term (multi-year) community disaster mental health monitoring, assessment, referral, outreach, and services are needed following major disasters like the 2011 Joplin, Missouri tornado.
